# Racial and Ethnic Demographic Reporting in Phase 2 Proton Therapy Clinical Trials: A Review

**DOI:** 10.14338/IJPT-22-00042.1

**Published:** 2023-05-15

**Authors:** Jonathan S. Fakhry, M. Juliana Pena, Ariel Pomputius, Fantine Giap, Raymond B. Mailhot Vega

**Affiliations:** 1 Department of Radiation Oncology, University of Florida College of Medicine, Jacksonville, FL, USA; 2 University of Florida Health Science Center Libraries, Gainesville, FL, USA

**Keywords:** proton therapy, race, demographics, phase 2 trial

## Abstract

**Purpose:**

Equitable inclusion of racial and ethnic participation in clinical trials is crucial to improving disparities in health care, especially for historically marginalized populations. Our study aims to describe the racial and ethnic demographics of patients enrolled in published phase 2 clinical trials involving proton therapy in the United States.

**Materials and Methods:**

Published manuscripts were identified in PubMed, Embase, World of Science, and Cochrane. Phase 2 trials evaluating proton therapy for US patients were included. For each article in the study, data were collected comprising authors, title, and publication year, and clinical trial numbers were verified. Additional data included tumor site, primary institution, sample size, reported race/ethnicity, and raw number/percentile of race/ethnicity. Preferred Reporting Items for Systematic Reviews and Meta-Analysis (PRISMA) guidelines were used.

**Results:**

Overall, 970 titles were identified; 636 remained after duplicate screening, and 75 full-text articles were assessed. We identified 38 eligible manuscripts for inclusion comprising 2648 patients. Only 15 (39%) of the publications reported race/ethnicity. Of these, 8 (21%) and 10 (26%) documented Hispanic or Black trial participants, respectively; however, only 6 (16%) documented trial participation for both Hispanic and Black patients. Of the 1409 patients with a documented race/ethnicity, 89.0% (n = 1254) were non-Hispanic white, 5.3% (n = 75) were Black, and 2.2% (n = 31) were Hispanic. Other and unknown race/ethnicity comprised the remaining patients (3.5%; n = 49).

**Conclusion:**

We identified underreporting of demographic data in published phase 2 proton therapy trials, which unfortunately mirrored underreporting for cancer drug clinical trials. We also noted dramatic Black and Hispanic patient underrepresentation across the trials in which race and ethnicity are reported. Findings highlight the urgent need to identify and address barriers to proton therapy trials for Black and Hispanic patients ensuring clinical trials in radiation oncology are representative of the patients seen in clinical practice.

## Introduction

The role of race and ethnicity within medical and health research has been highly controversial. While using these demographics may be potentially harmful in clinical algorithms and health-based risk scores because of the oversimplification of racial dichotomies, including these variables is important for identifying health disparities and determining the generalizability of a study [1, 2]. There is a contemporary and historic quandary with evidence across medical specialties demonstrating the underrepresentation of Black, Hispanic, and indigenous/native American patients in clinical trials in the United States. This lack of parity may lead to clinical practices that can be detrimental to the health of patients. For example, a recent study found that occult hypoxemia is more common in Black patients compared with white patients and is associated with increased mortality in an ICU setting based on lower sensitivity of pulse oximetry for hypoxemia in patients with darker skin tones [3].

Regarding cancer biology, it is understood that genetic ancestry is the more appropriate biologic variable to seek when the terms “race” and “ethnicity” are used. A common example is the higher risk of triple-negative breast cancer in women of African descent [4]. Regarding ethnicity, the term “Hispanic” comes from a census definition from the 1970s. In contrast, studies on genetic ancestry have demonstrated that heterogeneous representations of indigenous, Black, and Caucasian genetic ancestries are mainly based on the geographic regions from which an individual’s ancestors immigrated [5].

Because of previous confusion over what the terms race and ethnicity signify, the International Committee of Medical Journal Editors (ICMJE) developed 3 recommended guidelines for published research: (1) authors should “define how they determined race or ethnicity and justify their relevance”; (2) studies should include “representative populations into all study types and at a minimum provide descriptive data for these and other relevant demographic variables”; and (3) “if any demographic characteristics that were collected are not reported, the reason should be stated” [6]. Including race and ethnicity in research is vital both to provide transparency concerning the representativeness of the data and to identify potential inequalities within the health care system. Unfortunately, even though clear guidelines have been written on reporting race and ethnicity in research, they are often not reported as the guidelines recommend.

The use of proton radiation therapy (PT) has increased substantially within the past decade because of its benefits and decreased risks compared with traditional forms of photon beam therapy [7]. As a result, publications centered around PT have become increasingly important in managing clinical decision-making and outcomes. However, the cost of PT and its limited geographical availability create an increased burden for patients compared with traditional radiation therapy [8]. Past publications have demonstrated a demographic disparity in the use of PT; there is a positive correlation between its use for white patients, patients of a younger age, and patients of a higher socioeconomic status [9]. In addition, research has shown that Black patients are less likely to receive PT than white patients, especially when PT is the guideline-recommended treatment modality for the patient’s cancer type [10]. Other studies have similarly demonstrated that being a Black patient and of lower socioeconomic status are factors associated with a lower likelihood of receiving PT [11, 12].

If current scientific advancements are to benefit the population, it is imperative that physicians work to close these gaps and provide these beneficial treatments to population groups lacking equitable health care. Publications involving PT must then be based on heterogeneous and represented demographic groups; thus, the purpose of our study is to comprehensively investigate the inclusion of demographics in phase 2 PT studies through a systematic literature search of research publications. In addition, we aim to investigate the composition of enrollees of all demographic groups represented in phase 2 clinical trials.

## Materials and Methods

The goal of our systematic literature search was to identify all peer-reviewed articles relating to phase 2 trials pertaining to proton therapy, conducted in the United States, to determine the reporting and composition of race and ethnicity within the finalized manuscripts. A systematic literature search was conducted on July 27, 2021, to identify articles in the PubMed/MEDLINE, Embase, Web of Science, and Cochrane Database of Systematic Reviews databases. Terms such as “proton therapy,” “proton radiotherapy,” “particle beam radiation therapy,” and “proton radiation therapy” were used to identify studies published on the topic of phase 2 trials within proton therapy (**Supplemental Table 1**). Searches were limited to English-language publications (articles from US-only clinical studies would later be parsed out). The searches did not have a publication date limit.

Covidence, a systematic review management tool (Company name, City, State spelled out), was used to collect and screen eligible articles for the study. Two independent investigators (M.J.P., J.S.F.) performed title, abstract, and full-text screening to select eligible articles. A separate investigator (F.G.) served as a tiebreaker in cases where the 2 initial investigators screened an article differently. Studies were excluded per the following criteria: duplicate study, not a phase 2 study, not a US study, not a proton therapy–related study, or not a full research paper. In addition, any abstract-only papers, reviews, commentaries, or poster presentations were excluded. Of the articles collected, the following information was extracted in a data collection sheet: author, title, clinical trial number, publication year, PubMed identifier (PMID), tumor site, institution, location, sample size, reporting data/ethnicity, and raw number/percentile of race/ethnicity. Data on demographics were collected directly from the published articles as described by the authors. If a research article did not disclose the racial composition of the study cohort within the publication itself, it was disqualified. Differences in the descriptive categorization of racial groups were grouped and analyzed by the researchers for analytics. For example, African American and Black ethnicities were classified as “Black” for our study.

To determine comparison groups for the demographics described within the study articles we analyzed, we first attempted to relate the epidemiological data of oncologic diseases from the same institution that treated the type of cancer identified in the respective proton therapy publication. Of note, 3 of the 15 articles that reported race were multi-institutional studies. However, because of the lack of published reporting of race and ethnicity data in medical oncology, equivalent studies could not be used for comparison. As a solution, we subsequently used US census data from the corresponding cities where the studies took place to compare the patient populations reported in each research article. For example, in an article where Chicago was the study location, we used 2020 US census data from Cook County, where Chicago is located, to determine the demographic composition of the area. Counties were chosen instead of city populations to include a diversity of various geographical areas, including suburbs and smaller towns.

## Results

A total of 636 articles were reviewed. Of 38 eligible studies, only 15 reported demographics (39%), as noted in **Figure**. The combined percentage of white patients enrolled in the 15 studies was 83.7% (**Table 1**). The individual percentage range across studies based on geographical location was between 75% and 92% (**Table 2**). Five of 15 studies did not report the representation of Black-identifying patients in their published demographics (**Table 3**). Of the 10 studies that recorded representation of Black-identifying patients, there were 72 Black patients out of 1108 participants, representing an enrollment of 5.1% (**Table 1**). The individual percentage range across studies was 0% to 14%. None of the included studies reported indigenous/Native American patients as a demographic. Seven of 15 studies did not mention or report Latino/Hispanic patients as a demographic. The total percentage of patients that identified as Hispanic or Latino across the studies that reported ethnicity was 2.2% (**Table 1**). Of the 15 studies that reported race, 12 also reported gender (notably, manuscripts that studied sex-preferential cancers, e.g., breast and prostate cancers, were the only ones that did not report gender) (**Table 4**). When comparing each study’s cohort to the city where the study occurred, white patients were overrepresented in all 15 studies by a surplus range between 29.7% and 40.1%. Black/African Americans were underrepresented in a range between 11.7% and 38.6%, and Hispanics/Latinos were underrepresented in a range between 10% and 39.2% (**Table 2**).

**Table 1.** Cumulative number of research participants in all studies categorized by US census demographic racial categories.

**Race identified by US census^a^**	**Total number of participants^b^ (%)**
White alone	1179 (83.7)
Black or African American	72 (5.1)
American Indian and Alaskan Native	0 (0)
Asian	2 (0.14)
Native Hawaiian and other Pacific Islander	0 (0)
Two or more races	0 (0)
Hispanic or Latino	31 (2.2)

a These racial categories are based on US census reporting.

b Discrepancies in total number may be accounted for by the categorization that each individual study used and the inclusion of the term “other” as part of their reporting.

**Figure.**
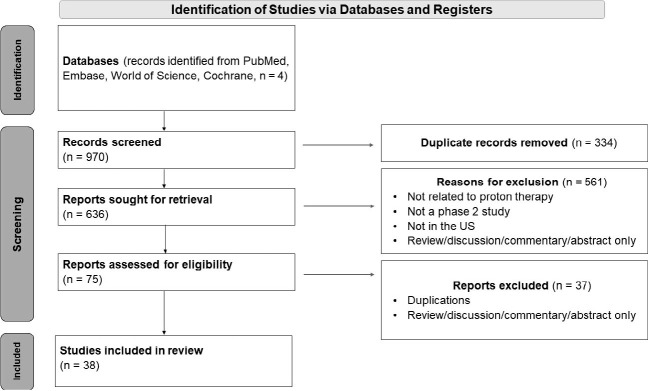
PRISMA diagram illustrating the studies identified, screened, eligible, and included for analysis in the study.

**Table 2.** Comparison of study population according to the location of county associated with the city to 2022 US census data.^a^

**Study Statistics**	**Population**				
	**Houston, TX**	**Philadelphia, PA**	**Boston, MA**	**Jacksonville, FL**	**Gainesville, FL**
Number of studies in location	5	1	2	3	1
Total number of participants	440	184	148	447	22
Participants identifying as white (%)	394 (89.5)	139 (75.0)	135 (91.2)	412 (92.0)	20 (90.9)
White population in county of studied location (%)	69.0	44.4	61.3	60.0	69.7
Participants identifying as black/African American (%)	12 (2.7)	28 (15.0)	1 (0.7)	28 (6.3)	2 (9.0)
Black/African American population in county of studied location (%)	20.3	43.6	24.4	31.1	20.7
Participants identifying as Hispanic/Latino (%)	23 (5.2)	0 (0)	0 (0)	6 (1.3)	0 (0)
Hispanic/Latino population in county of studied location (%)	44.4	15.9	23.8	11.3	11.0
Participants identifying as Asian^b^ (%)	7 (1.6)	1 (0.5)	2 (1.4)	0 (0)	0 (0)
Asian population in county of studied location (%)	7.4	8.0	9.5	5.1	6.2

a Three studies were not included because they were multi-institutional and spanned various cities.

b Asian was not included because most studies placed this ethnicity in the category “other.”

**Table 3.** Cumulative percentage of race represented in eligible studies (N = 15).

**Race included in the articles**	**Number of articles using that category (%)**
Asian	6 (40)
Black or African American	10 (66.6)
Hispanic	7 (46.6)
Other	6 (40.0)
White	15 (100.0)
Unknown	2 (13.3)

**Table 4.** Participant data by primary tumor site of eligible studies.

**Tumor site**	**Number of studies**	**Male (n)**	**Female (n)**	**Black/African American, n (%)**	**Asian, n (%)**	**Hispanic/Latino, n (%)**	**White, n (%)**
Brain	2	69	57	3 (2.4)	1 (0.8)	6 (4.8)	110 (87.0)
Lung	4	44	31	4 (5.0)	DNR	2 (2.7)	66 (88.0)
Prostate	2	DNR	DNR	63 (11.0)	1 (0.2)	5 (0.9)	490 (87.0)
Liver	1	100	64	4 (4.8)	2 (2.2)	1 (1.2)	76 (91.6)
Esophageal	1	97	10	2 (1.9)	1 (0.9)	11 (10.3)	98 (91.6)
Oropharyngeal	1	129	18	DNR	DNR	DNR	134 (91.0)
Breast	1	DNR	DNR	7 (7.0)	5 (5.0)	5 (5.0)	88 (88.0)
Muscle	1	27	30	DNR	DNR	DNR	50 (88.0)
Bone/cartilage	1	37	14	1 (2.0)	DNR	1 (2.0)	49 (96.0)
Multiple locations	1	96	50	1 (1.0)	2 (2.2)	DNR	82 (92.0)

Abbreviations: n, number; DNR, data not reported

Of the 15 eligible studies, a total of 7 racial/ethnic categories were reported, with each study using a different nominal categorization of the same race (**Table 5**). For example, 2 studies used the term African American when identifying their population cohort, while 8 used the term Black. However, none of the trials stated the process of racial and ethnic designation in their respective papers.

**Table 5.** List of various nominal racial categorizations and frequency of use in studies (N = 15)^a^

**Race terminology used**	**Number of articles using that category**
African American^b^	2
Asian	6
Black^b^	8
Hispanic	7
Other	6
Unknown	2
White	15

a Data aim to demonstrate the discrepancy of specific nominalization of races that were used in each individual study to identify their study population.

b Authors of the same paper used different categorizations of race.

## Discussion

First, our study demonstrates the overwhelming prevalence of underreporting of racial demographics in phase 2 clinical studies related to proton therapy. While the original intent was to evaluate race and ethnicity across all eligible trials, the number of phase 2 trials that did not report these important patient variables was striking. Furthermore, of those eligible trials that reported race and ethnicity, we can corroborate the barrier of minority population under-enrollment, a disparity not exclusive to clinical trials of cancer patients [13, 14]. The lack of unification of racial demographics among published works also appears prevalent in our reviewed studies. This deficiency is owing to the lack of defining categorization of race and ethnicity in studies despite the outlined US census characterization. Particularly harrowing is our inability to comment on Native American representation, as no included study reported any participation of patients of that demographic.

Indeed, a recent study by Nogueira et al [9] indicates that Black patients were less likely to receive proton therapy compared with white patients when that treatment was recommended over photon-based radiation therapy [9]. Whereas Nogueira et al sought to compare recommended treatment with completed treatment between 2 demographic categories, the present study investigates the enrollee composition for all demographic groups represented in phase 2 clinical trials.

The strength of our study is the use of multiple medical and scientific research databases, which allowed our study to analyze the most qualifying publications across a variety of institutions and journals. We used 4 major databases (PubMed, World of Science, Cochrane, and Embase), which helped ensure that we investigated most, if not all, publications that fit our criteria. Furthermore, using 3 separate investigators to vote on the inclusion criteria for the studies did much to ensure that all the relevant research articles were included in our systematic review.

While this work demonstrates an unacceptable underreporting of race and ethnicity in the published literature, it is important to acknowledge the limitations of this study. Such limitations include the relatively few publications related to proton radiation therapy, a product of the relatively recent introduction of this therapy for patients compared with photon-related treatments. In addition, the scarcity of proton radiation centers poses an additional bottleneck in the volume of studies within the field.

Another limitation of our study is the use of US census data when comparing population demographics to the study cohorts. Considering that all included publications in our study that reported racial demographics indicated that the studies took place at an academic institution, it is reasonable to assume that these tertiary care centers accepted their patients from larger geographic areas. This potentially augmented the difference between the participant population and the demographics of the city where the study occurred. This difference may also be compounded by the relative rarity of proton therapy centers within a given geographical area, such that many people may have had to travel even farther distances to receive treatment. In addition, 3 of the 15 articles included in this analysis were multi-institutional studies, further limiting the authors’ ability to make a valid comparison of racial demographics within a geographic area. A solution to account for the difference between city representation and health care centers would be to evaluate the data from each individual clinic; unfortunately, none of these data are publicly available for review.

Another limitation of this study is the potential for variations in cancer incidence rates based on race and ethnicity, with various studies highlighting this phenomenon. For example, the incidence of thyroid cancer in Asian and Pacific Islander patients is significantly lower than that of white and Black patients [15]. Within lung cancer, Black males continue to be disproportionally affected by squamous cell lung cancer and at the same time are diagnosed with more aggressive and malignant tumors than white males [16]. Prostate cancer incidence is also higher in Black patients compared with white patients [17]. The racial and ethnic differences in incidence could be owing to a variety of factors, including, but not limited, to social determinants of health and cultural or biological factors. Nonetheless, these discrepancies are an important consideration when analyzing the demographics of research participants.

In addition, the ambiguity in the methodology of racial demographics reporting posed another limitation to this study. For example, we could not determine whether a study used self-reporting of racial demographics (if self-reporting methods were used in some publications, it would account for the diversity of nominal categorizations we observed). Nevertheless, with *JAMA*’s new racial reporting guidelines, this problem should be alleviated in the future [18]. We encourage readers to use this *JAMA* article as a resource when considering how to report race and ethnicity appropriately.

This study ultimately highlights the lack of race reporting within research, which can lead to downstream effects in clinical translation. Heterogeneity and proper representation of population groups strengthen the generalizability and accuracy of data. Given differing socioeconomic inequalities, genetic predisposition, and the general lack of trust among minority patients within the current health care system [19, 20], a larger effort to include and report racial demographics is necessary for establishing reliable data that can be generalized to multiple demographic domains. *JAMA* recently recognized the importance of reporting demographics, encouraging research coordinators to include race in publications [18, 21]. Unfortunately, recruiting participants from historically marginalized backgrounds remains challenging, with reports of both implicit and explicit bias in excluding minority patients from cancer trials [22]. In addition, a lack of participation in cancer trials overall may stem from a dearth of community outreach to patients from historically marginalized backgrounds. This is especially vexing in light of one large meta-analysis reporting that minority patients are enthusiastic about participating in clinical trials, with consent rates approaching 60% when asked to enroll as opposed to the 2% to 5% we see nationally [23]. Along those lines, one large retrospective review demonstrated that belonging to a minority group or having low socioeconomic status did not affect the enrollment rate after screening patients diagnosed with glioma, indicating a problem associated with lack of screening and consent in these cohorts [24].

Furthermore, it is important to acknowledge the systemic barriers to proton beam treatment. Proton therapy participation can be limited by a variety of factors, including the ability to cover the cost and travel to a nearby center, as well as insurance type and status to cover the treatment. Therapy can cost 3 to 6 times as much as standard radiation treatment, largely owing to the complexity of the equipment and the sophistication of maintenance required to keep the machines running properly [25]. These financial limitations could disproportionately affect the ability of populations with low socioeconomic status in the area to receive proper treatment and be enrolled in studies that use these resources.

The importance of developing clinical trials that represent all Americans has been underscored nationally, with National Cancer Institute evaluations including these metrics in its standards for evaluating cancer clinics. With such an important focus, successful strategies to improve the equitable representation of these populations have been reported time and again. Such efforts typically begin with an awareness of biases and target measures to enroll a more diverse participant population. This includes the engagement of local community groups and private clinics related to health care, as well as partnerships with local stakeholders for minority patients; the latter is critical given the justifiable mistrust such communities may have based on the historical harm they have endured with medical research [26, 27]. Fortunately, the successful implementation of using public spaces (such as town halls) and having a presence at local community events to promote research studies in underrepresented communities has been demonstrated. However, implicit bias may still manifest as an unawareness on the part of clinical trial investigators with respect to the equitable representation of historically excluded populations, with a direct correlation between higher scores on implicit bias testing and lower quality of care [28].

We provide this call to action to all medical literature and clinical trial stakeholders, journals, authors, investigators, and health care centers to ensure that (1) active and actionable strategies to address and ensure equitable representation of historically excluded populations exist and (2) patient demographics are reported as a minimum when reporting outcomes.
