# Supplementary material for: Racial and Ethnic Demographic Reporting in Phase 2 Proton Therapy Clinical Trials: A Review
Source: Int J Part Ther. 2023 May 15;10(1):51–8. doi: 10.14338/IJPT-22-00042.1 (PMC10563665; doi:10.14338/IJPT-22-00042.1)
Supplement: Supplementary file 1 [file ijpt-10-01-03_s01.docx]

| **Supplemental Table 1**. Database Search Strategies | | | |
| --- | --- | --- | --- |
| Number | Database | Searches | Results |
| 1 | Embase | ('proton therapy'/exp OR 'proton beam therapy':ti,ab OR 'proton radiation therapy':ti,ab OR 'proton radiotherapy':ti,ab OR 'proton therapy':ti,ab OR 'proton beam radiotherapy':ti,ab OR 'particle therapy'/de OR 'particle beam radiation therapy':ti,ab OR 'particle beam radiotherapy':ti,ab OR 'particle beam therapy':ti,ab OR 'particle radiation therapy':ti,ab OR 'particle radio-therapy':ti,ab OR 'particle radiotherapy':ti,ab OR 'particle therapy':ti,ab) AND ('phase 2 clinical trial'/exp OR 'clinical trial, phase 2':ti,ab OR 'phase 2 clinical study':ti,ab OR 'phase 2 clinical trial':ti,ab OR 'phase 2 study':ti,ab OR 'phase 2 trial':ti,ab OR 'phase ii clinical study':ti,ab OR 'phase ii clinical trial':ti,ab OR 'phase ii study':ti,ab OR 'phase ii trial':ti,ab) AND [english]/lim | 347 |
| 2 | PubMed | (("proton therapy"[Title/Abstract] OR "particle therapy"[Title/Abstract] OR "proton radiotherapy"[Title/Abstract] OR "proton beam therapy"[Title/Abstract] OR "proton beam radiotherapy"[Title/Abstract] OR "particle beam therapy"[Title/Abstract] OR "particle radiation therapy"[Title/Abstract]) OR (("Proton Therapy"[Mesh]) OR "Heavy Ion Radiotherapy"[Mesh])) AND (("phase 2 clinical trial"[Title/Abstract] OR "phase 2"[Title/Abstract] OR "phase II"[Title/Abstract] OR "phase two"[Title/Abstract]) OR ("Clinical Trials, Phase II as Topic"[Mesh])) | 46 |
| 3 | Web of Science | ("proton therapy" OR "particle therapy" OR "proton radiotherapy" OR "proton beam therapy" OR "proton beam radiotherapy" OR "particle beam therapy" OR "particle radiation therapy” OR "Heavy Ion Radiotherapy") AND (("phase 2 clinical trial" OR "phase 2" OR "phase II" OR "phase two") | 57 |
| 4 | Cochrane | ("proton therapy" or "particle therapy" or "proton radiotherapy" or "proton beam therapy" or "proton beam radiotherapy" or "particle beam therapy" or "particle radiation therapy" or "heavy ion radiotherapy") AND ("phase 2 clinical trial" OR "phase II clinical trial" OR "phase 2" OR "phase two") | 201 |
